# Peripheral blood cell anomalies in COVID-19 patients in the United Arab Emirates: A single-centered study

**DOI:** 10.3389/fmed.2022.1072427

**Published:** 2022-12-15

**Authors:** Noha Mousaad Elemam, Iman M. Talaat, Fatehia A. Bayoumi, Dima Zein, Ramy Georgy, Abdalrahman Altamimi, Noura Alkhayyal, Alaa Habbal, Feda Al Ali, Alaa ElKhider, Abdallah Ahmed, Salah Abusnana, Riyad Bendardaf

**Affiliations:** ^1^Clinical Sciences Department, College of Medicine, University of Sharjah, Sharjah, United Arab Emirates; ^2^Sharjah Institute of Medical Research, University of Sharjah, Sharjah, United Arab Emirates; ^3^Medcare Hospital Sharjah, Sharjah, United Arab Emirates; ^4^Nursing Department, University Hospital Sharjah, Sharjah, United Arab Emirates; ^5^Medical Diagnostic Imaging Department, University Hospital Sharjah, Sharjah, United Arab Emirates; ^6^Medical Laboratory Department, University Hospital Sharjah, Sharjah, United Arab Emirates; ^7^Internal Medicine Department, University Hospital Sharjah, Sharjah, United Arab Emirates

**Keywords:** CO-RADS, COVID-19, lymphocytes, neutrophils, peripheral smears, UAE

## Abstract

**Introduction:**

In this study, we aimed at exploring the morphologic and quantitative abnormalities in the peripheral blood counts of coronavirus disease 2019 (COVID-19) patients.

**Methods:**

A cohort of 131 COVID-19 patients was recruited at University Hospital Sharjah (UHS), UAE. Their peripheral blood smears were examined for morphological evaluation. Also, their clinical laboratory investigations and radiological findings were retrieved from the medical records. Our cohort consisted of 63 males and 68 females with an age of 63.6 ± 18.6 years.

**Results:**

The presence of atypical lymphocytes was observed in around 80% of the recruited COVID-19 patients. Further, monocytes with toxic cytoplasmic vacuoles were identified in 55% of the cases. Neutrophil-associated changes, including pseudo-Pelger-Huët, bands, and long nuclear endoplasm, were reported in around 25–35% of the patients. RBCs associated changes such as microcytic and hypochromic RBCs, as well as targetoid, dacrocytes, ovalocytes, echinocytes/burr cells, and schistocytes, were described. According to disease severity, RBCs chromicity was found to be significantly different between stable and critical patients. COVID-19 patients with CO-RADS 5 showed a similar change in RBCs as well as a decrease in the neutrophils with hypogranular cytoplasm.

**Conclusion:**

Peripheral blood smear assessment in COVID-19 patients could provide information about the disease state and pulmonary involvement.

## Introduction

Coronavirus disease 2019 (COVID-19) remains a global pandemic, caused by severe acute respiratory syndrome coronavirus 2 (SARS-CoV-2), which affects multiple organs (1). In the United Arab Emirates (UAE), the first case of COVID-19 was identified on 29 January 2020 (2). The most common symptoms in COVID-19 patients include fever, fatigue, cough, and dyspnea (3). However, some cases could be asymptomatic carriers while others fall into mild, moderate, and severe categories. In critical COVID-19 patients, fatal acute respiratory distress syndrome (ARDS) occurs, leading to intensive care unit (ICU) admission (4, 5).

It was found that COVID-19 pathogenesis was associated with an activation of the immune system and subsequent immune dysregulation (6). A major clinical feature of COVID-19 was neutrophilia with concomitant lymphopenia, that were linked to the severity of the disease (7). Despite the known quantitative abnormalities in the peripheral blood, little is known about the morphologic changes in circulating blood cells in COVID-19 (8). Such changes could aid in the diagnosis of COVID-19 and management decisions in COVID-19 patients. The reported abnormalities in peripheral blood smears include a range of atypical lymphocytes, acquired Pelger-Huët anomaly, and fetus-shaped neutrophils (9). Furthermore, abnormal platelets and red blood cells (RBCs) morphology were also reported in peripheral blood of COVID-19 infected patients, thus inducing coagulopathies and malfunction of oxygen carrying capacity (10). In this study, we aim to explore the morphologic and quantitative abnormalities in the peripheral blood counts of COVID-19 patients recruited to a single center in the UAE.

## Subjects and methods

This is a retrospective study conducted on 131 COVID-19 patients that were recruited at University Hospital Sharjah (UHS), UAE. Our cohort was composed of 63 males and 68 females, aged 63.6 ± 18.6 years (mean ± SD). The cases were diagnosed based on a positive nasopharyngeal swab result using reverse transcriptase-polymerase chain reaction (RT-PCR). Out of 270 patients that were admitted from July 2020 to July 2021, 131 COVID-19 patients were selected as they were not previously vaccinated for SARS-CoV-2. The study was approved by the Ethics and Research Committee of UHS (UHS-HERC-035-03052020).

Peripheral blood samples were collected from COVID-19 patients in EDTA sterile vacutainers, after which peripheral blood smears were prepared, and laboratory investigations were performed. These tests included complete and differential blood such as platelets, white blood cells (WBCs), neutrophils, lymphocytes, monocytes count, oxygen saturation and hemoglobin that were done using Sysmex XN 20 Hematology Analyzer (Sysmex, Germany). Also, prothrombin time (PT), international normalized ratio (INR), activated partial thromboplastin time (aPTT), and D-dimer were measured using STA Compact Max 3 (Stago, France). Moreover, lactate dehydrogenase (LDH) and C-reactive protein (CRP) were measured using Atellica^®^ CH 930 Analyzer (Siemens Healthineers, Germany), while procalcitonin and ferritin were measured Atellica^®^ IM 1300 Analyzer (Siemens Healthineers, Germany). All these tests are summarized in Table 1. Blood films were prepared and stained using the Leishman stain standard protocol. The smears were examined under the light microscope (Olympus BX43, Japan) by a hematopathologist for morphological evaluation, and images were captured using the digital camera (Olympus SC50, Japan). The evaluation was done blindly in terms of laboratory investigations.

**TABLE 1:** Clinical and demographic of 131 coronavirus disease 2019 (COVID-19) patients recruited in this study.

Total COVID-19 cases	*n* = 131	
Gender	63 males and 68 females	
Age (years) (Mean ± SD)	63.6 ± 18.6	
	**Normal range**	**Mean ± SD**
Oxygen saturation	≥95	91.9 ± 5.3
Hemoglobin (g/L)	13.0–17.5	11.9 ± 2.2
Platelet count (×10^9^/L)	125–350	262.2 ± 172.1
White blood cell count (×10^9^/L)	3.5–9.5	9 ± 17.6
Neutrophil count (×10^9^/L)	1.8–6.3	7.9 ± 17.6
Lymphocyte count (×10^9^/L)	1.1–3.2	1.3 ± 1.3
Monocyte count (×10^9^/L)	0.1–0.6	0.6 ± 0.7
Lactate dehydrogenase (U/L)	135–225	302.6 ± 192.4
Prothrombin time (seconds)	11.5–14.5	14.8 ± 2.2
International normalized ratio – INR	0.8–1.2	1.1 ± 0.3
Activated partial thromboplastin time (seconds)	29–42	38.4 ± 7
D-dimer (μg/mL)	<0.5	2.5 ± 5.5
Procalcitonin (ng/mL)	0.02–0.05	4.9 ± 42.7
C-reactive protein (CRP) (mg/L)	<1	83.2 ± 73.4
Ferritin (μg/L)	30–400	462.5 ± 511.1

Radiological evaluation was done using chest X-ray and high-resolution computed tomography (CT) scans, followed by an assessment using the COVID-19 Reporting and Data System (CO-RADS) as a standardized assessment of pulmonary involvement of COVID-19 (11). The CO-RADS classification is described in Table 2.

**TABLE 2:** COVID-19 Reporting and Data System (CO-RADS) interpretation with level of suspicion for pulmonary involvement of COVID-19 infection and its corresponding CT findings.

CO-RADS	Level of suspicion for pulmonary involvement of COVID-19 infection	CT findings
1	Highly unlikely	Normal or non-infectious abnormalities
2	Unlikely	Abnormalities consistent with infections other than COVID-19
3	Equivocal	Unclear whether COVID-19 is present
4	Probable	Abnormalities suspicious of COVID-19
5	Highly likely	Typical COVID-19
6	PCR proven	–

Values represent mean ± SD for the continuous variables, or percentage relative to the total number of patients in each group for the categorical variables. Statistical analysis was performed using GraphPad Prism 6 software (GraphPad Software, San Diego, CA, USA). Chi-square test was used for the comparison between the categorical variables. *P*-value < 0.05 was considered statistically significant.

## Results

Our cohort was composed of 131 patients that were proven to be COVID-19 positive by RT-PCR of the nasopharyngeal swabs. Their quantitative hematologic abnormalities were documented along with the microscopic examination of the peripheral smears to include various anomalies such as changes in WBCs, RBCs, and platelets.

**FIGURE 1:**
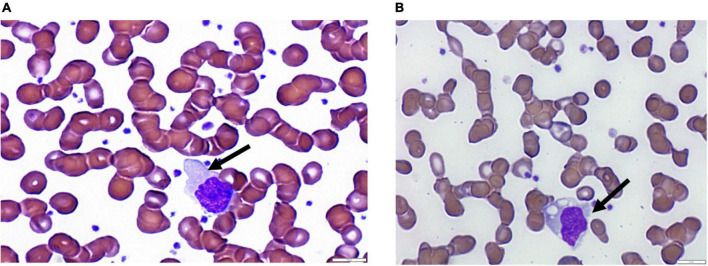
Images showing peripheral changes in lymphocytes and monocytes. **(A)** Atypical lymphocytes and **(B)** monocytes with vacuolated cytoplasm.

The most common reported morphologic finding was the presence of atypical lymphocytes in around 80% of the COVID-19 patients (Figure 1A). This was followed by the presence of monocytes with toxic cytoplasmic vacuoles in 55% of the cases. As shown in Figure 1B, activated monocytes were observed showing prominent cytoplasmic vacuolization and few granules. Also, the nuclei were large, having fine chromatin with nuclear blebbing.

**FIGURE 2:**
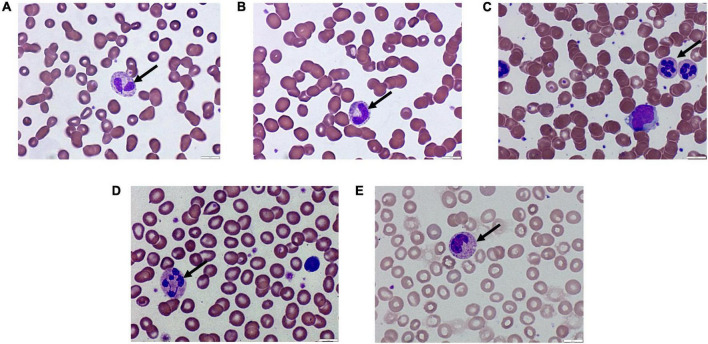
Images showing peripheral changes in neutrophils. **(A)** Pseudo-Pelger-Huët, **(B)** bands, **(C)** hypersegmented, **(D)** long nuclear cytoplasm, and **(E)** fetus-shaped nuclei in neutrophils.

Neutrophil-associated changes, including pseudo-Pelger-Huët (Figure 2A), bands (Figure 2B), hypersegmented (Figure 2C), and long nuclear endoplasm (Figure 2D), were observed in some of the recruited COVID-19 patients. Such neutrophilic-associated changes were reported in around 25–35% of the patients, as shown in Table 3. Also, C-shaped, fetus-like nuclei were noted with aberrant nuclear projections (Figure 2E).

**TABLE 3:** Changes in the white blood cells (WBCs) count and morphological anomalies including lymphocytes, monocytes, and neutrophils.

	Frequency (*n*)	Percent (%)
**WBC changes**		
Leucocytosis	4	3.1
Leukopenia	4	3.1
**Lymphocyte changes**		
Lymphocytosis	2	1.5
Lymphopenia	26	19.8
Atypical lymphocytes	105	80.2
Granular lymphocyte cytoplasm	5	3.8
Basophilic cytoplasm lymphocytes	6	4.6
Plasmacytoid lymphocytes	0	0.0
**Monocyte changes**		
Monocytosis	4	3.1
Monocytes with vacuoles	72	55.0
**Neutrophils changes**		
Neutrophilia	19	14.5
Neutropenia	2	1.5
Pseudo-Pelger-Huët	33	25.2
Bands	34	26.0
Long nuclear endoplasm	33	25.2
Fetus-shaped nucleus	30	22.9
Macrogranular neutrophil cytoplasm	12	9.2
Karyorrehxis neutrophils	3	2.3
Drumstick nucleus	2	1.5
Hypogranular neutrophil cytoplasm	48	36.6

**FIGURE 3:**
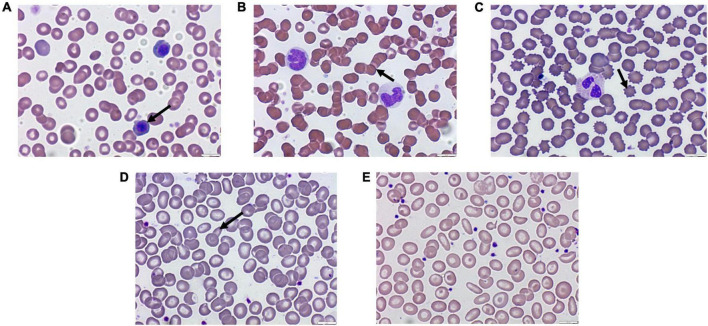
Images showing peripheral changes in red blood cells (RBCs). **(A)** Normoblasts, **(B)** rouleaux, **(C)** acanthocytes, **(D)** mushroom-like, and **(E)** anisopoikilocytosis, ovalocytes, schistocytes, and targetoid cells.

Regarding platelet counts, the mean number was within the normal range in COVID-19 patients, which aligns with the coagulation parameters (PT, INR, and aPTT) that were almost in their normal ranges. Around half of the COVID-19 patients presented with normocytic RBCs along with anisocytosis and hyperchromicity (Table 4). Other common RBCs associated changes included erythrocytopenia and microcytic RBCs as well as targetoid, dacrocytes, ovalocytes, echinocytes/burr cells, and schistocytes (Figures 3A–E).

**TABLE 4:** Changes in the platelets and red blood cells (RBCs) count and their associated morphological anomalies.

	Frequency (*n*)	Percent (%)
**Platelets changes**		
Platelets thrombocytosis	17	13
Platelets thrombocytopenia	15	11.5
**RBCs changes**		
Normocytic RBCs	66	50.4
Anisocytosis	65	49.6
Hypochromic RBCs	55	42
Targetoid	47	35.9
Normochromic RBCs	44	33.6
Dacrocytes	42	32.1
Erthrocytopenia	35	26.7
Ovalocytes	35	26.7
Microcytic RBCs	34	26
Echinocytes/Burr cells	34	26
Schistocytes	33	25.2
Bite cells	28	21.4
Stomatocytes	24	18.3
Acanthocytes/Spur	24	18.3
Rouleaux RBCs	23	17.6
Mushroom RBCs	21	16
Polycythemia	10	7.6
Polychromasia	5	3.8
Normoblasts	4	3.1
Macrocytic RBCs	2	1.5

**FIGURE 4:**
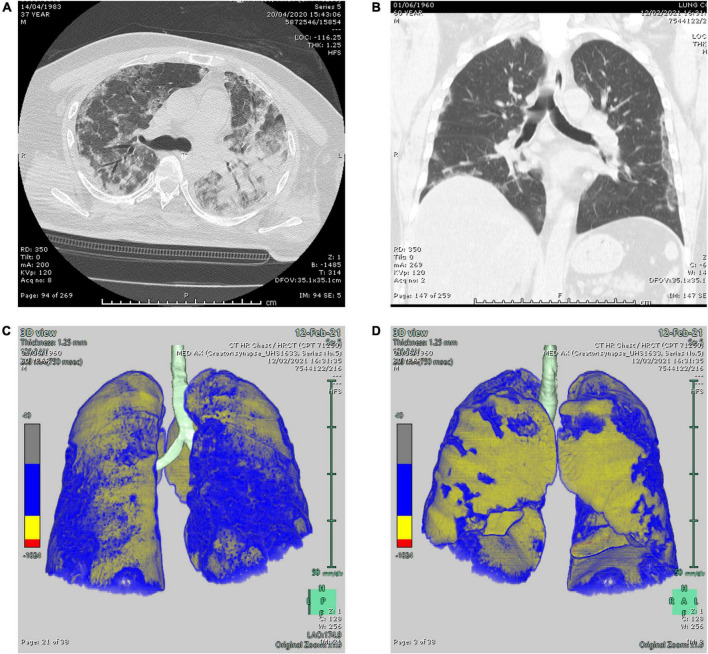
Two representative computed tomography (CT) lung window images. **(A)** Axial and **(B)** coronal lung window CT images showing bilateral scattered ground-glass attenuation opacities with an area of pneumonic consolidation at the left lower lobe (CO-RADS 5). **(C,D)** 3D reconstructed images of the lungs showing bilateral subpleural ground-glass attenuation opacities (CO-RADS 5) with 27.6% parenchymal high attenuation areas in both lungs (calculation performed on Fujifilm Synapse 4D PACS dedicated application).

The COVID-19 patients were classified into different groups according to their CO-RADS score. Almost 77% of the patients fell into the CO-RADS 5 category (Figure 4), indicating pulmonary involvement and a high probability of COVID-19 infection before confirmatory tests by qRT-PCR (Table 5).

**TABLE 5:** Radiological assessment of COVID-19 patients using the CO-RADS.

	Frequency (*n*)	Percent (%)
CO-RADS 1	17	13.2
CO-RADS 2	6	4.7
CO-RADS 3	4	3.1
CO-RADS 4	3	2.3
CO-RADS 5	99	76.7
Presence of acute respiratory distress syndrome	18	13.8

In order to search for a relation between morphological changes in peripheral blood cells and disease severity, COVID-19 patients were classified into stable (patients not requiring ICU admission, *n* = 48) and critical (ICU admitted patients, *n* = 83) groups. No statistical significance was found between the two groups except for RBCs changes and platelets’ thrombocytosis (Table 6). There was a significant increase in the percentage of patients showing normocytic (*p* = 0.0301), normochromic (*p* = 0.0246), with a significant decrease in the patients’ microcytic (*p* = 0.0109), and hypochromic RBCs (*p* = 0.0158) in critical ICU-admitted patients. Furthermore, stable COVID-19 patients showed higher levels of platelets’ thrombocytosis compared to critical COVID-19 patients (*p* = 0.0209).

**TABLE 6:** Count and morphological changes in WBCs, platelets, and RBCs between stable and critical COVID-19 patient groups.

	Stable (*n* = 48)		Critical (*n* = 83)		
		
	Frequency	Percentage	Frequency	Percentage	*P*-value
**WBC changes**					
Leucocytosis	2	4.2	2	2.4	0.2867
Leukopenia	2	4.2	2	2.4	0.2867
**Neutrophils changes**					
Neutrophilia	5	10.4	14	16.9	0.1562
Neutropenia	1	2.1	1	1.2	0.3464
Pseudo-Pelger-Huët	11	22.9	22	26.5	0.3242
Bands	11	22.9	23	27.7	0.2732
Long nuclear endoplasm	10	20.8	23	27.7	0.1911
Fetus shaped nucleus	9	18.8	21	25.3	0.195
Macroganular neutrophil cytoplasm	5	10.4	7	8.4	0.3523
Karyorrehxis neutrophils	0	0.0	3	3.6	0.0913
Drumstick nucleus	1	2.1	1	1.2	0.3464
Hypoganular neutrophil cytoplasm	19	39.6	29	34.9	0.2975
**Lymphocyte changes**					
Lymphocytosis	1	2.1	1	1.2	0.3464
Lymphopenia	10	20.8	16	19.3	0.4148
Atypical lymphocytes	39	81.3	66	79.5	0.4054
Granular lymphocyte cytoplasm	3	6.3	2	2.4	0.1345
Basophilic cytoplasm lymphocytes	2	4.2	4	4.8	0.4317
Plasmacytoid lymphocytes	0	0.0	0	0.0	–
**Monocyte changes**					
Monocytosis	2	4.2	2	2.4	0.2867
Monocytes with vacuoles	24	50.0	48	57.8	0.1927
**Platelets changes**					
Platelets thrombocytosis	10	20.8	7	8.4	0.0209*
Platelets thrombocytopenia	5	10.4	10	12.0	0.3888
**RBCs changes**					
Normocytic RBCs	19	39.6	47	56.6	0.0301*
Normochromic RBCs	11	22.9	33	39.8	0.0246*
Erthrocytopenia	13	27.1	22	26.5	0.4713
Polycythemia	5	10.4	5	6.0	0.1808
Normoblasts	1	2.1	3	3.6	0.3118
Polychromasia	1	2.1	4	4.8	0.2155
Microcytic RBCs	18	37.5	16	19.3	0.0109*
Macrocytic RBCs	1	2.1	1	1.2	0.3464
Hypochromic RBCs	26	54.2	29	34.9	0.0158*
Rouleaux RBCs	9	18.8	14	16.9	0.3925
Anisocytosis	20	41.7	45	54.2	0.0831
Targetoid	18	37.5	29	34.9	0.3842
Stomatocytes	12	25.0	12	14.5	0.0664
Mushroom RBCs	9	18.8	12	14.5	0.2594
Acanthocytes/Spur	7	14.6	17	20.5	0.2002
Ovalocytes	14	29.2	21	25.3	0.315
Dacryocytes	18	37.5	24	28.9	0.1552
Schistocytes	14	29.2	19	22.9	0.2127
Echinocytes/Burr cells	9	18.8	25	30.1	0.0763
Bite cells	12	25.0	16	19.3	0.2207

*p < 0.05, significant results.

Another possible classification was to divide COVID-19 patients into those belonging to CO-RADS 5 group (*n* = 99) versus others (*n* = 30). There was an observed significant decrease in the neutrophils showing hypogranular cytoplasm in CO-RADS 5 group (*p* = 0.049). Also, there was a significant increase in the percentage of patients showing normocytic (*p* = 0.0431) and normochromic (*p* = 0.0135) RBCs in the CO-RADS 5 group. Further, there was a significant decrease in the percentage of patients showing ovalocytes in the CO-RADS 5 group (*p* = 0.0194, Table 7).

**TABLE 7:** Count and morphological changes in WBCs, platelets, and RBCs between COVID-19 patients with CO-RADS 5 and other CO-RADS.

	CO-RADS 5 (*n* = 99)		Other CO-RADS (*n* = 30)		
		
	Frequency	Percentage	Frequency	Percentage	*P*-value
**WBC changes**					
Leucocytosis	3	3.0	1	3.3	0.4666
Leukopenia	2	2.0	2	6.7	0.0992
**Neutrophils changes**					
Neutrophilia	16	16.2	3	10.0	0.2021
Neutropenia	1	1.0	1	3.3	0.1834
Pseudo-Pelger-Huët	27	27.3	6	20.0	0.2119
Bands	27	27.3	6	20.0	0.2119
Long nuclear endoplasm	26	26.3	7	23.3	0.3737
Fetus shaped nucleus	24	24.2	6	20.0	0.315
Macroganular neutrophil cytoplasm	8	8.1	4	13.3	0.1928
Karyorrehxis neutrophils	3	3.0	0	0.0	0.1673
Drumstick nucleus	1	1.0	1	3.3	0.1834
Hypogranular neutrophil cytoplasm	33	33.3	15	50.0	0.049*
**Lymphocyte changes**					
Lymphocytosis	2	2.0	0	0.0	0.2163
Lymphopenia	17	17.2	9	30.0	0.0625
Atypical lymphocytes	78	78.8	25	83.3	0.2933
Granular lymphocyte cytoplasm	3	3.0	2	6.7	0.183
Basophilic cytoplasm lymphocytes	4	4.0	1	3.3	0.4302
Plasmacytoid lymphocytes	0	0.0	0	0.0	–
**Monocyte changes**					
Monocytosis	2	2.0	2	6.7	0.0992
Monocytes with vacuoles	56	56.6	15	50.0	0.2633
**Platelets changes**					
Platelets’ thrombocytosis	11	11.1	5	16.7	0.2093
Platelets’ thrombocytopenia	11	11.1	4	13.3	0.3697
**RBCs changes**					
Normocytic RBCs	54	54.5	11	36.7	0.0431*
Normochromic RBCs	38	38.4	5	16.7	0.0135*
Erthrocytopenia	24	24.2	10	33.3	0.161
Polycythemia	7	7.1	3	10.0	0.2996
Normoblasts	3	3.0	1	3.3	0.4666
Polychromasia	3	3.0	2	6.7	0.183
Microcytic RBCs	23	23.2	10	33.3	0.1333
Macrocytic RBCs	2	2.0	0	0.0	0.2163
Hypochromic RBCs	38	38.4	16	53.3	0.073
Rouleaux RBCs	16	16.2	7	23.3	0.1843
Anisocytosis	49	49.5	15	50.0	0.4807
Targetoid	35	35.4	11	36.7	0.4477
Stomatocytes	17	17.2	6	20.0	0.3615
Mushroom RBCs	14	14.1	7	23.3	0.1161
Acanthocytes/Spur	16	16.2	8	26.7	0.0976
Ovalocytes	21	21.2	12	40.0	0.0194*
Dacryocytes	28	28.3	13	43.3	0.0605
Schistocytes	24	24.2	9	30.0	0.2633
Echinocytes/Burr cells	27	27.3	7	23.3	0.3339
Bite cells	21	21.2	7	23.3	0.4025

*p < 0.05, significant results.

Since gender plays a critical role in the COVID-19 pathogenesis (11, 12), it was interesting to explore if there is any difference in the peripheral blood anomalies between males (*n* = 63) and females (*n* = 68). There was a statistical significance in the chromicity of RBCs, where males showed a higher significant percentage of normochromic (*p* = 0.0093), with a concomitant decrease in the percentage of hypochromic RBCs (*p* = 0.0054, Table 8).

**TABLE 8:** Count and morphological changes in WBCs, platelets, and RBCs between male and female COVID-19 patients.

	Male (*n* = 63)		Female (*n* = 68)		
		
	Frequency	Percentage	Frequency	Percentage	*P*-value
**WBC changes**					
Leucocytosis	1	1.6	3	4.4	0.2032
Leukopenia	2	3.2	2	2.9	0.3906
**Neutrophils changes**					
Neutrophilia	13	20.6	6	8.8	0.074
Neutropenia	0	0.0	2	2.9	0.1249
Pseudo-Pelger-Huët	15	23.8	18	26.5	0.4562
Bands	17	27.0	17	25.0	0.3125
Long nuclear endoplasm	17	27.0	16	23.5	0.2271
Fetus shaped nucleus	13	20.6	17	25.0	0.4025
Macroganular neutrophil cytoplasm	6	9.5	6	8.8	0.438
Karyorrehxis neutrophils	1	1.6	2	2.9	0.1903
Drumstick nucleus	0	0.0	2	2.9	0.1249
Hypoganular neutrophil cytoplasm	23	36.5	25	36.8	0.3703
**Lymphocyte changes**					
Lymphocytosis	0	0.0	2	2.9	0.1249
Lymphopenia	16	25.4	10	14.7	0.2869
Atypical lymphocytes	52	82.5	53	77.9	0.4305
Granular lymphocyte cytoplasm	2	3.2	3	4.4	0.2195
Basophilic cytoplasm lymphocytes	3	4.8	3	4.4	0.4405
Plasmacytoid lymphocytes	0	0.0	0	0.0	–
**Monocyte changes**					
Monocytosis	1	1.6	3	4.4	0.2032
Monocytes with vacuoles	38	60.3	34	50.0	0.1391
**Platelets changes**					
Platelets’ thrombocytosis	7	11.1	10	14.7	0.0794
Platelets’ thrombocytopenia	9	14.3	6	8.8	0.2715
**RBCs changes**					
Normocytic RBCs	33	52.4	33	48.5	0.0903
Normochromic RBCs	28	44.4	16	23.5	0.0093*
Erthrocytopenia	15	23.8	20	29.4	0.347
Polycythemia	6	9.5	4	5.9	0.438
Normoblasts	1	1.6	3	4.4	0.4228
Polychromasia	4	6.3	1	1.5	0.1415
Microcytic RBCs	16	25.4	18	26.5	0.0853
Macrocytic RBCs	2	3.2	0	0.0	0.3627
Hypochromic RBCs	19	30.2	36	52.9	0.0054*
Rouleaux RBCs	13	20.6	10	14.7	0.4025
Anisocytosis	27	42.9	38	55.9	0.45
Targetoid	20	31.7	27	39.7	0.2634
Stomatocytes	9	14.3	15	22.1	0.0767
Mushroom RBCs	11	17.5	10	14.7	0.4305
Acanthocytes/Spur	16	25.4	8	11.8	0.0819
Ovalocytes	21	33.3	14	20.6	0.3199
Dacryocytes	22	34.9	20	29.4	0.3896
Schistocytes	18	28.6	15	22.1	0.4727
Echinocytes/Burr cells	18	28.6	16	23.5	0.1161
Bite cells	13	20.6	15	22.1	0.2927

*p < 0.05, significant results.

## Discussion

This study highlights the quantitative and morphological changes in the peripheral blood cells of COVID-19 patients. To our knowledge, this is the first study to report these changes in the UAE, which has taken extraordinary precautionary measures to restrict the spread of COVID-19 and guarantee the safety of citizens. Furthermore, this study explored if there is an association between disease severity and peripheral blood anomalies.

The most common morphological anomalies in our cohort were atypical lymphocytes, large monocytes with vacuoles, and hypogranular neutrophil cytoplasm of the peripheral blood smears. Despite the small number of reports on the peripheral morphological anomalies associated with COVID-19 infection, our findings go in line with a study by Zhang et al. where large monocytes with vacuoles were observed in peripheral smears of COVID-19 patients (13). In addition, our observed morphological anomalies were consistent with the findings by Zini et al. that reported various peripheral morphological blood changes, specifically in the neutrophils (14). Further, the presence of atypical lymphocytes was highly found to be similar to the previous reports (15–17). In addition, granulocytes and particularly neutrophils showed a pseudo-Pelger-Huët anomaly affecting 25% of the recruited COVID-19 patients, both stable and critical cases, unlike the observed findings by Ahnach et al. (18). Similarly, another type of neutrophil-associated anomaly was the presence of hypogranular cytoplasm in more than 30% of the patients (18).

Despite the advances in COVID-19 research, little information about the morphological changes in peripheral blood smears of infected individuals and their association with patients’ clinical outcomes is still unknown. Thus, we were interested in correlating the difference in these peripheral anomalies in ICU-admitted and stable COVID-19 patients. An increase in the percentage of patients showing neutrophilia, neutrophils with pseudo-Pelger-Huët, bands, long nuclear endoplasm, and fetus shaped nucleus, as well as large vacuolated monocytes, was recognized in the critical/ICU admitted group; however, such changes did not reach statistical significance. An increase in the percentage of aforementioned anomalies was also observed in COVID-19 patients with a CO-RADS score 5 compared to others. On the other hand, upon the classification of COVID-19 patients according to the severity of the disease or CO-RADS, there was a decrease in the number of patients showing hypogranular neutrophilic cytoplasm, which could be a sign of pulmonary deterioration. On the contrary, a study by Gabr et al. reported that the abundance of peripheral morphological abnormalities was significantly associated with unfavorable clinical outcomes in COVID-19 patients (17).

A plethora of abnormalities associated with RBCs was previously described in COVID-19 patients (19, 20). Morphological changes in COVID-19 patients were detected, along with a comparison between stable and critical cases. There was a significant decrease in the percentage of COVID-19 patients in the critical group with microcytic and hypochromic RBCs, along with a significant increase in the percentage of patients with normocytic and normochromic RBCs. A similar pattern was observed in the classification of COVID-19 according to CO-RADS. Additionally, there was a concomitant decrease in hemoglobin concentration in stable COVID-19 patients (11.4 ± 2.3) compared to critical (12.2 ± 2.0) cases. Hence, this could be attributed to other factors such as ferritin concentration or other underlying chronic diseases in such patients. Further, such findings suggest that anemia could be linked to inflammation, a known manifestation of COVID-19 infection. This was further confirmed with a higher percentage of female COVID-19 patients presenting hypochromic RBCs, which supports previous findings by Bergamaschi et al. (21).

## Conclusion

To our knowledge, this is the first study in the UAE describing the morphological changes in the peripheral blood smears of COVID-19 patients and their association with disease severity. Peripheral blood smear assessment at the time of diagnosis in COVID-19 patients could provide information about the disease state and pulmonary involvement. One of the limitations of this study is the lack of information about the recruited patients’ mortality and the lack of peripheral smears of healthy controls for comparative investigation. Also, another limitation of this study is the lack of functional analysis of the aforementioned peripheral blood cells (including oxygen-carrying capacity of RBCs, ROS generation by leukocytes and immunoglobulin production by lymphocytes, phagocytosis capacity of macrophages/monocytes and leukocytes), that should be explored in future studies to further understand their role in the fight against SARS-CoV-2. Additionally, future studies could explore the association between antibody titers against SARS-CoV-2 proteins and hematological abnormalities that will aid in understanding the effect of infection and vaccinations on various blood cells.

## Data availability statement

The original contributions presented in this study are included in this article/supplementary material, further inquiries can be directed to the corresponding authors.

## Ethics statement

The studies involving human participants were reviewed and approved by the Ethics and Research Committee of University Hospital Sharjah in June 2020 (UHS-HERC-035-03052020). Written informed consent for participation was not required for this study in accordance with the national legislation and the institutional requirements.

## Author contributions

NE and IT designed the study, analyzed, interpreted the results, and wrote the original draft. FB interpreted the slides of the peripheral blood smears. RG assessed the radiological imaging of the patients. DZ, AAl, NA, AH, FA, AE, AAh, and SA recruited and collected the data of the patients. RB designed the study. FB, RG, and RB reviewed the manuscript. All authors read and approved the final manuscript.
